# Decoding the Gene Regulatory Network of Muscle Stem Cells in Mouse Duchenne Muscular Dystrophy: Revelations from Single-Nuclei RNA Sequencing Analysis

**DOI:** 10.3390/ijms241512463

**Published:** 2023-08-05

**Authors:** Yan Shen, Il-Man Kim, Yaoliang Tang

**Affiliations:** 1 Medical College of Georgia, Augusta University, Augusta, GA 30912, USA; yashen@augusta.edu; 2 Anatomy, Cell Biology, and Physiology, School of Medicine, Indiana University, Indianapolis, IN 46202, USA; ilkim@iu.edu

**Keywords:** cell metabolism, Duchenne muscular dystrophy, *dystrophin*, single-nuclear RNA sequencing (snRNA-seq), skeletal muscle-derived muscle stem cells

## Abstract

The gene *dystrophin* is responsible for Duchenne muscular dystrophy (DMD), a grave X-linked recessive ailment that results in respiratory and cardiac failure. As the expression of *dystrophin* in muscle stem cells (MuSCs) is a topic of debate, there exists a limited understanding of its influence on the gene network of MuSCs. This study was conducted with the objective of investigating the effects of *dystrophin* on the regulatory network of genes in MuSCs. To comprehend the function of *dystrophin* in MuSCs from DMD, this investigation employed single-nuclei RNA sequencing (snRNA-seq) to appraise the transcriptomic profile of MuSCs obtained from the skeletal muscles of *dystrophin* mutant mice (*DMD^mut^*) and wild-type control mice. The study revealed that the *dystrophin* mutation caused the disruption of several long non-coding RNAs (lncRNAs), leading to the inhibition of MEG3 and NEAT1 and the upregulation of GM48099, GM19951, and GM15564. The Gene Ontology (GO) enrichment analysis of biological processes (BP) indicated that the *dystrophin* mutation activated the cell adhesion pathway in MuSCs, inhibited the circulatory system process, and affected the regulation of binding. The study also revealed that the metabolic pathway activity of MuSCs was altered. The metabolic activities of oxidative phosphorylation (OXPHOS) and glycolysis were elevated in MuSCs from *DMD^mut^*. In summary, this research offers novel insights into the disrupted gene regulatory program in MuSCs due to *dystrophin* mutation at the single-cell level.

## 1. Introduction

*Dystrophin* plays a crucial role in linking the sarcomere and the extracellular matrix and stabilizing the *dystrophin*-associated glycoprotein complex during muscle contraction and relaxation [1]. In Duchenne muscular dystrophy (DMD), the mutation of the *dystrophin* gene results in progressive degeneration of the striated muscles, including cardiac and skeletal muscles that leads to the depletion of the regenerative potential of muscle stem cells (MuSCs) by repeated muscle loss–regeneration–muscle loss cycles [2,3]. Therefore, DMD can also be considered a stem cell disease.

The controversy surrounding the expression of *dystrophin* in MuSCs persists due to the proximity of satellite cells to myofibers, which normally express *dystrophin* in muscle sections [4]. Past research has posited that MuSCs do not express *dystrophin* and that only myotubes differentiated from MuSCs exhibit *dystrophin* expression [5,6,7]. Nonetheless, a recent study verified *dystrophin* expression in C2C12 cells, an immortal mouse MuSC line [8]. Moreover, another investigation demonstrated that a high-density plating of C2C12 cells led to increased *dystrophin* expression [9]. Jelinkova S. et al. [10] revealed that human pluripotent stem cells (hPSCs) express various *dystrophin* forms, and a lack of *dystrophin* expression in hPSCs could render the cells susceptible to *dystrophin*-related stem cell dysfunction. Our prior research also indicated that iPSC-derived MuSCs exhibit dystrophin expression on the cell membranes [2].

Dystrophin plays a crucial role in establishing the polarity and alignment of the mitotic axis, which is necessary for the correct progression of asymmetric stem cell divisions [4]. In mice with Mdx mutation, MuSCs have been observed to have various mitotic abnormalities. These abnormalities include atypical expression of phosphorylated Aurora kinase, amplification of centrosomes, and impaired kinetics of cell division [11,12].

Due to the heterogeneity of MuSCs, it is essential to examine the expression of *dystrophin* in individual MuSCs of muscle tissues. Single-nuclei RNA sequencing (SnRNA-seq) technology is an ideal approach to resolve this issue. In addition, there is limited knowledge about the impact of *dystrophin* deficiency on the gene program of MuSCs. A single cell-level comprehensive analysis of the transcriptome in individual MuSCs can help us to uncover the gene regulatory network that is controlled by dystrophin in MuSCs.

In order to investigate the heterogeneity and transcriptional dysregulation of DMD in MuSCs at the single-cell level, we conducted a re-analysis of a publicly available snRNA-seq database. This dataset was derived from a mouse model of DMD that harbors a CRISPR-Cas9-mediated deletion of exon 51 (∆Ex51) in the *dystrophin* gene. This mutation is known to be a prevalent cause of DMD in humans. In a previous study by Chemello et al. [13], it was demonstrated that the absence of functional *dystrophin* in ΔEx51 muscle leads to degeneration of muscle tissue, which is subsequently replaced by fibrotic tissue and characterized by inflammatory infiltration. Our snRNA-seq analysis revealed that skeletal muscle-derived MuSCs expressed the *dystrophin* gene. Through a systematic comparison of muscle-derived MuSCs from *dystrophin* mutant (*DMD^mut^*) mice and control mice, we identified key target genes of *dystrophin* in MuSCs and discovered that the *dystrophin* mutation significantly activated pathways involved in the cell adhesion and suppressed pathways involved in circulatory system process and regulation of binding. The snRNA-seq analysis also showed differences in the metabolic pathways of muscle-derived MuSCs from *DMD^mut^* mice compared to control mice. Overall, this study provides insight into the *dystrophin*-mediated gene regulation network in MuSCs.

## 2. Results

### 2.1. Single Cell Transcriptomics Reveals MuSC Clusters

**Figure 1:**
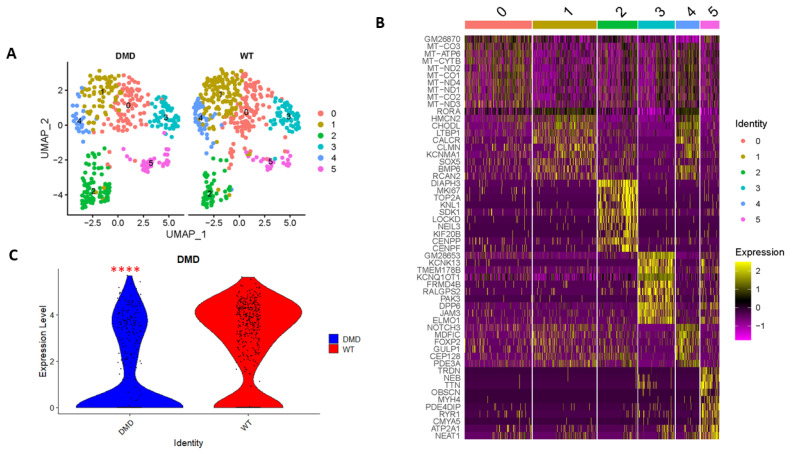
Single-nuclei RNA sequence analyses. (**A**) Split view of Uniform Manifold Approximation and Projection (UMAP) plot representation of the MuSCs from *DMD^mut^* and WT mouse samples. (**B**) Heatmap of the top 10 signature genes of six MuSC clusters. (**C**) The expression levels of *dystrophin* in MuSC population from *DMD^mut^* and WT mouse muscle samples were visualized in violin plot (**** *p* < 0.0001).

We conducted snRNA-seq analyses on muscle tissues obtained from *DMD^mut^* and control mice. We then focused on examining the presence of *dystrophin* in MuSCs by isolating MuSC clusters. Our findings indicate that there was a slightly higher number of MuSCs in *DMD^mut^* muscles (368 MuSCs out of 3937 muscle-derived cells, i.e., 9.3%) as compared to control muscles (443 MuSCs out of 6409 muscle-derived cells, i.e., 6.9%). This suggests that the *dystrophin* mutation-induced degeneration of myofibers may trigger MuSCs to proliferate and replace the damaged skeletal muscles. We also identified six subpopulations of MuSCs (cluster 0–5) (Figure 1A). The top 10 marker genes with the highest differential expression levels in each cell cluster are shown in Figure 1B and Table 1. Our analysis revealed that the expression of *DMD* was significantly lower in *DMD^mut^* MuSCs as compared to control MuSCs (Figure 1C).

### 2.2. Differential Gene Expression and Functional Enrichment Analysis of MuSCs in DMD^mut^ Versus Control Muscles

**Figure 2:**
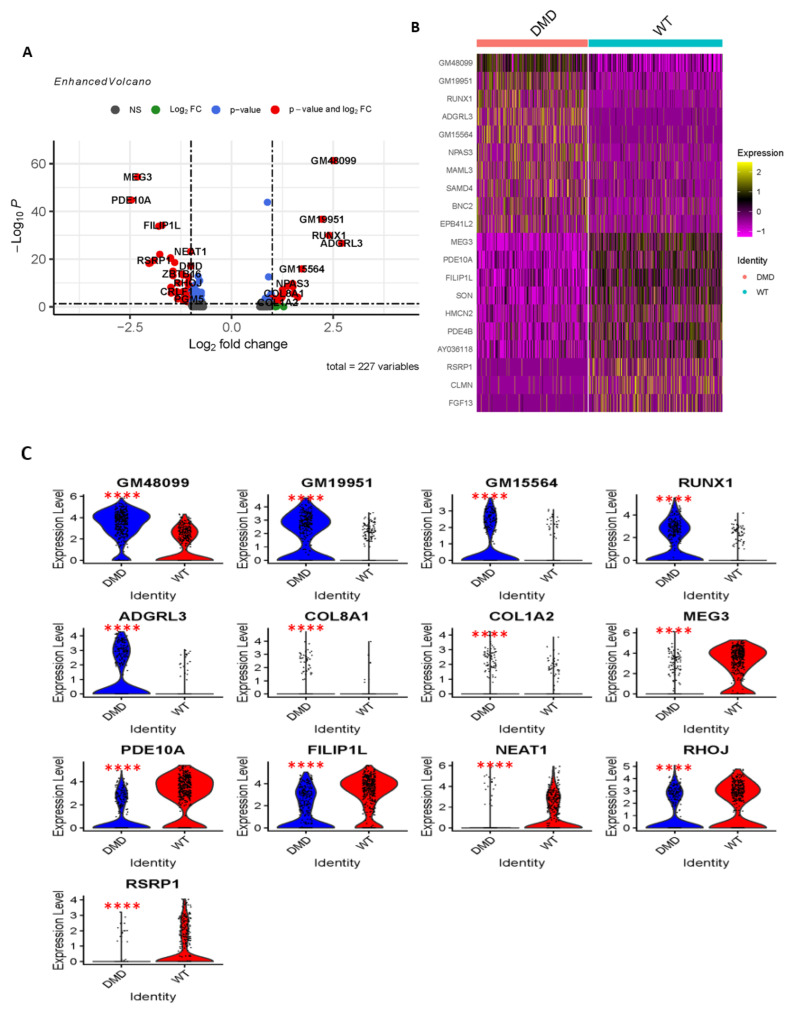
Differential gene expression in MuSCs from *DMD^mut^* versus control muscles. (**A**) Differential gene expression in MuSCs from *DMD^mut^* ^versus^ control muscles. Volcano plots were used to show the adjusted *p* values and log2 fold change values of the genes in MuSCs from *DMD^mut^* ^and^ wild-type control muscles. Differentially expressed genes (DEGs) are represented by red dots. The genes highlighted on the right side of the plot are upregulated in *DMD^mut^* MuSCs, while the genes on the left side are downregulated; (**B**) Heatmap of the top 10 signature genes between MuSCs from *DMD^mut^* versus control muscles; and (**C**) The expression levels of GM48099, GM19951, GM15564, RUNX1, ADGRL3, COL8A1, COL1A2, MEG3, PDE10A, FILIP1L, NEAT1, RHOJ, and RSRP1 in MuSC population from *DMD^mut^* and WT mouse muscle samples were visualized in violin plot (**** *p* < 0.0001).

**Figure 3:**
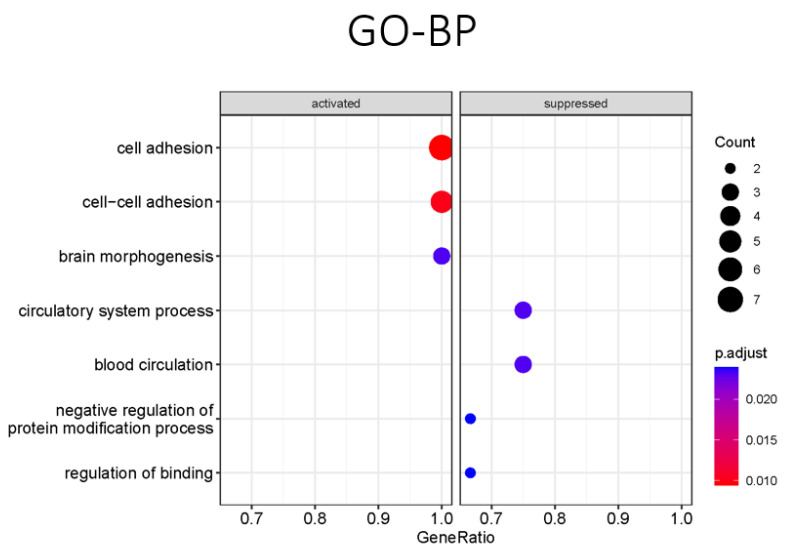
Functional enrichment analysis of MuSCs specific expression change of *DMD^mut^* and WT muscles. Dot plot representing the top four GO-BP pathways with the largest gene ratios in the order of gene ratio. The color gradient of dots represents the adjusted *p* values, while the size represents the number of genes in the significant DEG list associated with the GO-BP term.

We performed differential gene expression analysis to compare MuSCs in *DMD^mut^* and control muscle and found that *DMD^mut^* MuSCs had significantly elevated levels of various long noncoding RNAs (lncRNAs), including GM48099, GM19951, and GM15564. Additionally, *Runx1*, *Adgrl3*, *Npas3*, *Col8a1*, and *Col1a2* were also significantly upregulated in *DMD^mut^* MuSCs (Figure 2A–C). We conducted a GO enrichment biological process (BP) analysis using the clusterProfiler package and found that the three most significantly activated pathways in *DMD^mut^* MuSCs are related to cell adhesion, cell-cell adhesion, and brain morphogenesis (Figure 3). These findings highlight the important role of *dystrophin* in regulating lncRNAs, cell adhesion, and brain morphogenesis.

**Figure 4:**
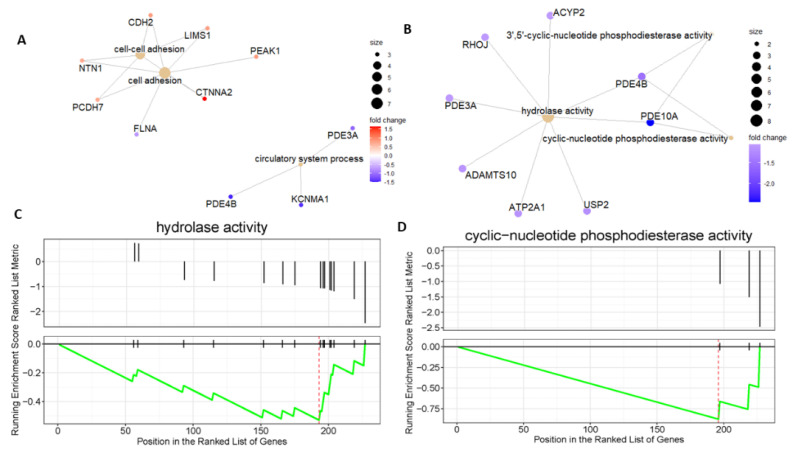
(**A**,**B**) The cnetplot depicts the linkages of genes and GO terms as a network. This is helpful to see which genes are involved in enriched pathways; (**C**,**D**) GSEA Plot of the Running Enrichment Score (green line) for a gene set as the analysis walks down the ranked gene list, including the location of the maximum enrichment score (the red line). The black lines in the Running Enrichment Score show where the members of the gene set appear in the ranked list of genes, indicating the leading edge subset.

The gene expression analysis revealed that a number of genes were significantly downregulated in *DMD^mut^* MuSCs, including two lncRNAs (MEG3 and NEAT1) and several other genes, such as *Pde10a*, *Filip1l*, *Rsrp1*, *Zbtb16*, *Rhoj*, *Crlf1*, and *Pgm5* (Figure 2A–C). The GO-BP analysis revealed that the pathways, which are most significantly suppressed in *DMD^mut^* MuSCs, are mainly related to the circulatory system process, blood circulation, negative regulation of protein modification process, and regulation of binding (Figure 3). To understand the complex relationship between the enriched pathways, we used the Cnetplot function. This allows visualization of the genes involved in the enriched pathways and genes belonging to annotation categories. Figure 4A shows the network of suppressed circulatory system process, which includes downregulated genes (*Pde3a*, *Pde4b*, and *Kcnma1*) and activated cell adhesion network and cell–cell adhesion network, which include upregulated genes (*Ctnna2*, *Cdh2*, *Lims1*, *Ntn1*, *Peak1*, and *Pcdh7*). Figure 4B shows the network of suppressed hydrolase activity, 3′, 5′-cyclic-nucleotide phosphodiesterase activity, and cyclic-nucleotide phosphodiesterase activity, which are indicated by downregulated genes (*Pde10a, Ped4b, Ped3a,* and *Rhoj*). To identify top pathway responsible for the observed phenotypes in *DMD^mut^* and control MuSCs, we performed the gene set enrichment analyses. The major gene set enriched in the *DMD^mut^* MuSCs compared to control MuSCs was classified for inhibited hydrolase activity and cyclic-nucleotide phosphodiesterase activity (Figure 4C,D).

### 2.3. Expression Analysis of Muscle Differentiation and Proliferation Genes in DMD Mutant MuSCs

**Figure 5:**
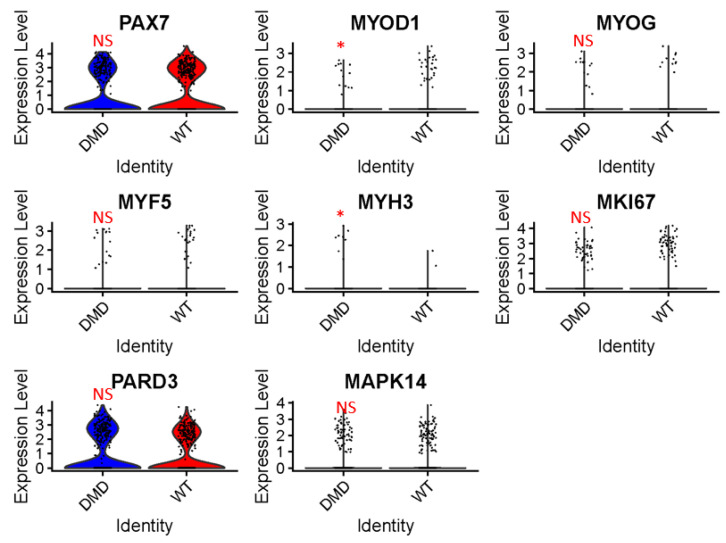
The expression levels of PAX7, MYOD1, MYOG, MYF5, MYH3, MKI67, PARD3, and MAPK14 in MuSC population from *DMD^mut^* and WT mouse muscle samples were visualized in violin plot (NS, not significant, * *p* < 0.05).

Although the GO-BP analysis did not identify pathways specifically associated with differentiation or proliferation, it is imperative to investigate the expression of major muscle differentiation and proliferation genes in the context of *DMD* mutant MuSCs and control MuSCs. This is particularly important considering the well-known impaired regenerative capacity of MuSCs in DMD [14]. To address this, we conducted a comparative analysis of the expression levels of key muscle stem cell differentiation genes, including PAX7, MYOD1, MYOG, MYF5, and MYH3, as well as the major proliferation gene MKI67, and the genes PARD3 and P38/MAPK14. The results, depicted in Figure 5, demonstrate that the expression of MYOD1 was significantly downregulated in *DMD* mutants, whereas the expression of MYH3 was upregulated. However, we did not observe significant differences in the mRNA expression of PAX7, MYOG, MYF5, MKI67, PARD3, and MAPK14 between the *DMD* mutant and control groups. These findings suggest that while MYOD1 and MYH3 expression is affected in *DMD* mutant MuSCs, the expression of other muscle differentiation and proliferation genes remains relatively unchanged. It is worth noting that the compromised repair capacity of MuSCs in DMD is closely associated with progressive MuSC senescence [15]. Therefore, it is important to consider the age of the mice from which the snRNA-seq dataset was obtained, as they were relatively young (4 weeks old). At this early stage, compensatory mechanisms may be in effect, and senescence-induced impairments in MuSC proliferation and differentiation potential might not be fully manifested.

### 2.4. Differential Metabolic Pathways of MuSCs within DMD^mut^ and Control Muscles

**Figure 6:**
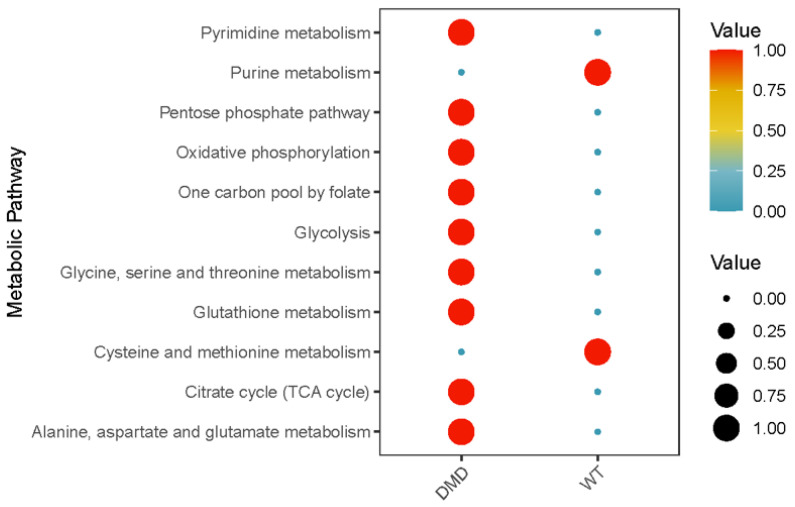
Dot plot illustration of the metabolic pathway analyses performed using “scMetabolism” R package (https://github.com/wu-yc/scMetabolism, accessed on 29 July 2023) for MuSCs from *DMD^mut^* and WT muscles.

We investigated the metabolic changes in MuSCs after *dystrophin* mutation using the “scMetabolism” package, which covers a wide range of metabolic pathways. The metabolic pathways were filtered, and the activity was compared between MuSCs from *DMD^mut^* and control muscles. The results indicated significant differences in multiple metabolic pathways: (1) During various stages of differentiation, MuSCs undergo dynamic metabolic reprogramming. Analysis of “scMetabolism” indicated that MuSCs from *DMD^mut^* muscles had higher activities of oxidative phosphorylation (OXPHOS), citric acid cycle (TCA cycle), glycolysis metabolism, and pentose phosphate pathway (PPP) activities compared to MuSCs from control muscles (Figure 6); (2) Purines and pyrimidines are fundamental building blocks of nucleotides, which are crucial for cell proliferation. Proliferative cells require nucleotides, including purines and pyrimidines for synthesizing cellular components [16]. When there is a dysregulation in purine and pyrimidine metabolism, there is an increase in DNA damage and cell turnover [17]. Analysis of “scMetabolism” indicated that MuSCs from *DMD^mut^* muscles had reduced purine metabolism activities and increased pyrimidine metabolism activities, indicating dysregulation in purine and pyrimidine metabolism after the *dystrophin* mutation (Figure 6); and (3) Our analysis of amino acid metabolic pathways reveals that MuSCs from individuals with *dystrophin* mutations exhibit heightened activity in glutathione metabolism, whereas displaying lower activity in cysteine and methionine metabolism in comparison to MuSCs from healthy muscle (Figure 6). Glutathione (GSH) is essential for cellular defense mechanisms against oxidative stress-induced damage, functioning through glutathione peroxidase (GPX) to maintain redox homeostasis in cells [18,19]. Homocysteine (Hcy) serves as an intermediary amino acid, being metabolized into methionine or cysteine [20]. Hcy has been shown to lead to oxidative stress and altered mitochondrial function [21]. Additionally, Hcy promotes stem cell differentiation [22]. Recent research has demonstrated that individuals with *dystrophin* mutations exhibiting poorer muscle function demonstrate greater oxidative damage and lower antioxidant function than those with better muscle function [23]. Therefore, it would be intriguing to investigate whether *dystrophin* mutations provide protection to MuSCs within an oxidative stress environment.

## 3. Discussion

In this study, we examined snRNA-seq data derived from the skeletal muscles of *DMD^mut^* mice and control mice. The findings confirmed the expression of the *dystrophin* gene in normal MuSCs, and its expression was reduced in *DMD^mut^* mice. Furthermore, snRNA-seq revealed that the most active pathways in *DMD^mut^* MuSCs were related to cell adhesion, whereas the most suppressed pathways were linked to circulatory system processes, blood circulation, negative regulation of protein modification, and binding regulation. In addition, we noted dysregulation of lncRNAs, including upregulation of several lncRNAs (e.g., GM48099, GM19951, GM15564) and downregulation of others (MEG3 and NEAT1). The *dystrophin* mutation also affected major energy metabolism pathways in MuSCs, with increased activities observed in OXPHOS, citric acid cycle (TCA cycle), glycolysis metabolism, and PPP, which could potentially enhance MuSC-mediated muscle regeneration.

The complex known as the *dystrophin* glycoprotein complex (DGC) is composed of five classes of proteins (dystroglycans, syntrophins, dystrobrevins, sarcoglycans, and sarcospan). Its main function is to connect the extracellular matrix to the actin cytoskeleton, enabling three important processes: maintaining the structural stability of the plasma membrane, regulating ion levels within cells, and facilitating transmembrane signaling [24]. Apart from being expressed in skeletal and cardiac muscle cells, *dystrophin* has also been found to be expressed in neurons, astrocytes, and glial cells of both the central and peripheral nervous systems [25,26], as well as in the retina [27] and the kidneys [28,29]. Additionally, *dystrophin* is present in vascular smooth muscle cells (VSMCs), where it plays a role in ion channel function. Mutations in *dystrophin* in VSMCs lead to significantly increased expression of KCNQ5 and RYR2, potentially resulting in low blood pressure in DMD patients [30]. *Dystrophin* is also expressed in endothelial cells (ECs), and *DMD* mutations lead to the suppression of SPTBN1 and the upregulation of several long noncoding RNAs (lncRNAs), including GM48099, GM19951, and GM15564, in ECs [31].

PDEs, or cyclic nucleotide phosphodiesterases, are a group of phosphohydrolases that selectively break down the 3’ cyclic phosphate bonds of adenosine and/or guanosine 3′,5′ cyclic monophosphate, thereby regulating the cellular levels of the second messengers, cAMP and cGMP [32]. Our analysis of snRNA-seq gene ontology pathways showed that the *dystrophin* mutation in MuSCs resulted in the suppression of pathways associated with circulatory system processes and blood circulation, in which *Pde3a* and *Pde4b* were implicated. Additionally, we observed significant downregulation of *Pde10a* expression in *DMD^mut^* MuSCs. *Pde10a* is the only gene identified in the *Pde10* family and serves as a secondary messenger in numerous signaling pathways and physiological processes [33]. Recent research suggests that inhibiting *Pde10a* reduces cancer cell growth and promotes cell cycle arrest and apoptosis by suppressing β-catenin and RAS signals in cancer cells [34], indicating that *Pde10a* plays a critical role in cell proliferation. However, another recent study found that specifically suppressing *Pde10a* enhances motor and muscle function, as well as vascular function and long-term survival in sapje-like zebrafish larvae. This study also revealed that *Pde10a* may modulate *Pitpna* expression in DMD patient-derived myogenic cells [35]. The conflicting findings could be explained by *Pde10a*’s distinct roles in muscle stem cells and differentiated muscle cells because cell proliferation is crucial for MuSC-mediated muscle regeneration, whereas *Pde10a* inhibition-induced *Pitpna* downregulation might positively impact muscle function.

New findings have provided insights into the molecular mechanisms through which lncRNAs govern skeletal muscle mass and function. These mechanisms encompass transcriptional regulation, fiber-type switching, and the proliferation of skeletal muscle cells [36]. LncRNAs participate in the regulation of diverse biological processes via multiple mechanisms. They are transcribed by RNA polymerase II in a manner that is influenced by the cellular environment and exhibit similar characteristics to mRNAs, including 5′ caps, polyA tails, and splice sites. The presence of polyA tails in these lncRNAs allows their detection through Chromium Single Cell 3’ 10x Genomics single-cell RNA sequencing, a technique that captures RNAs based on their 3′-biased polyA tails [31,37]. The role of these lncRNAs in the pathophysiology of dystrophic MuSCs remains to be determined. Our analysis of snRNA-seq data revealed that the *dystrophin* mutation affects the expression of multiple lncRNAs in MuSCs, including significantly upregulated expression of *Gm48099*, *Gm19951*, and *Gm15564*, as well as significantly downregulated expression of maternally expressed gene 3 (*Meg3*) and nuclear-enriched abundant transcript 1 (*Neat1*). *Gm48099*, *Gm19951*, and *Gm15564* are also upregulated in *DMD^mut^* endothelial cells and skeletal muscle cells, suggesting that dysregulation of these lncRNAs is conserved across different cell lines. However, the functional role of these lncRNAs and how the *dystrophin* mutation causes their upregulation remains unexplored. *Meg3*, an imprinted maternally suppressive lncRNA, was reported as a crucial regulator that inhibits myoblast proliferation and promotes myoblast differentiation in porcine satellite cells [38]; thus, the downregulation of *Meg3* in *DMD^mut^* MuSCs may facilitate MuSC proliferation. *Neat1* is an architectural lncRNA, and its expression increases during C2C12 differentiation [39]. Inhibition of *Neat1* was reported to delay skeletal muscle regeneration following cardiotoxin (CTX) injection in the gastrocnemius muscle, resulting in reduced satellite cell numbers [36,39] and suggesting that downregulation of *Neat1* in *DMD^mut^* MuSCs may hinder MuSC differentiation and proliferation. Our snRNA-seq data analysis also showed a slightly higher percentage of MuSCs in *DMD^mut^* muscles than in control muscles, indicating activated MuSCs in *DMD^mut^* skeletal muscles.

The mutation of *dystrophin* gene leads to a significant increase in the expression of *Runx1*. This protein is a sequence-specific DNA binding transcription factor and is known to be associated with the stem cell function of various tissues. It plays a critical role in regulating stem cell fate in MuSCs, mesenchymal stem cells, hematopoietic stem cells, hairy follicle stem cells, mammary epithelial stem cells, and neural stem cells [40]. Studies have shown that *Runx1* regulates the balance between proliferation and differentiation in MuSCs during muscle regeneration. When *Runx1* is deleted in primary MuSCs, they exhibit lower rates of proliferation and higher rates of spontaneous differentiation. Conversely, overexpression of *Runx1* delays differentiation and reduces the number of multinucleated myofibers [41,42].

Mutation of *dystrophin* significantly increased the expression of Adhesion G Protein-Coupled Receptor L3 (*Adgrl3*), Neuronal PAS Domain Protein 3 (*Npas3*), *Col8a1*, and *Col1a2*. *Adgrl3* is an adhesion GPCR involved in cell–cell and cell–extracellular matrix interactions [43]. *Adgrl3* forms a trimeric complex with fibronectin leucine-rich transmembrane protein 3 (*Flrt3*) and *Unc5*, which provide cell adhesion and glutamatergic synapse development [44]. *Npas3* belongs to a group of transcription factors called basic helix-loop-helix (bHLH) PAS family and has been associated with psychiatric and neurodevelopmental disorders [45]. Although the exact function of *Npas3* in MuSCs is not yet established, studies have shown that knocking down *Npas3* can maintain the stemness of radial glial cells and increase the proliferation of neural progenitor cells found in the VZ/subventricular zone (SVZ) [45]. This implies that an elevated expression of *Npas3* in *DMD^mut^* MuSC could lead to a loss of stemness in these cells. When *dystrophin* is absent, it causes a cyclical process of muscle fiber degeneration and regeneration. This is accompanied by an increase in collagen content and fibrosis on a histological level [46]. Collagen cross-linking is a structural change that occurs in dystrophic muscles, and it has been observed to increase passive muscle stiffness in fibrotic models such as the mdx mouse model of DMD [47]. Our analysis of the snRNA-seq dataset revealed that the expression of both *Col2a2* and *Col8a1* was significantly elevated in MuSCs.

Our analysis also revealed that several other crucial genes involved in MuSC function, including Filamin A Interacting Protein 1 Like (*Filip1l*), Arginine And Serine Rich Protein 1 (*Rsrp1*), Zinc Finger And BTB Domain Containing 16 (*Zbtb16*), Ras Homolog Family Member J (*Rhoj*), Cytokine Receptor Like Factor 1 (*Crlf1*), and Phosphoglucomutase 5 (*Pgm5*) were significantly downregulated in *DMD^mut^* MuSCs compared to control cells. The activation of Wnt/β-catenin signaling has been observed in the muscles of DMD patients [48]. Studies have reported a link between the reduction of *Filip1l* and upregulation of Wnt/β-catenin signaling. This signaling pathway has been implicated in the proliferation of cancer cells, inflammation, and fibrosis within the tumor microenvironment [49]. Therefore, the downregulation of *Filip1l* in *DMD^mut^* ^MuSC^ may contribute to the activation of Wnt signaling in the muscles of DMD patients. It is unclear what role *Zbtb16* (also referred to as PLZF) and RHOJ play in MuSC function. However, research suggests that ZBTB16 plays a critical role in promoting self-renewal of spermatogonial stem cells in mice [50]. On the other hand, *Rhoj*, a small Rho GTPase, is known to act as a crucial regulator, promoting resistance to a wide range of chemotherapeutic agents in epithelial-to-mesenchymal transition (EMT) of tumor cells [51].

Our gene ontology pathway analysis using snRNA-seq data revealed that the *dystrophin* mutation led to increased activation of cell adhesion pathways in MuSCs, including cell adhesion, cell–cell adhesion, and adherens junction. The upregulation of cell adhesion signaling in MuSCs may indicate that they play an active role in muscle cell regeneration in *DMD^mut^* mice. This is because MuSCs require an adhesion substrate to attach to the basal lamina of myofibers and establish a connection between the MuSC and mature muscle. This connection is crucial for driving myogenic signaling during the regeneration process that follows skeletal muscle damage [52,53].

MuSCs remain inactive in their designated niche until they are stimulated by external signals, such as injury, that prompt them to enter the cell cycle. The fate of MuSCs is intricately linked to the amount of energy generated by their mitochondria. As MuSCs progress through various differentiation stages, their metabolic pathways undergo dynamic changes. Quiescent MuSCs mostly rely on mitochondrial OXPHOS to produce ATP and do not depend heavily on glycolysis. However, activated MuSCs/muscle progenitor cells (MPC) increase their glycolysis rate during activation and proliferation [54]. As MPCs begin to differentiate, they suppress glycolysis in favor of OXPHOS, which is essential for the final differentiation of muscle fibers. Additionally, intermediates of the tricarboxylic acid (TCA) cycle act as cofactors for enzymes that carry out epigenetic remodeling processes, such as histone acetylation and DNA methylation, which significantly affect the self-renewal, commitment, and differentiation of MuSCs [55]. Our snRNA-seq metabolic analysis shows that the *dystrophin* mutation causes an increase in OXPHOS, TCA cycle, and glycolysis in MuSCs. The upregulation of both glycolysis and OXPHOS activities could contribute to the activation and proliferation of MuSCs and the differentiation of MPCs into myofibers in *DMD^mut^* mice. This finding is particularly relevant since DMD is characterized by constant myofiber degeneration triggered by contractions, followed by a regenerative response that involves the activation of quiescent MuSCs, the proliferation of resulting MPCs, and the terminal differentiation and fusion of MPCs into myofibers [56].

## 4. Materials and Methods

### 4.1. SnRNA-Seq Datasets

The snRNA-seq data (barcodes, features, and matrix of gene expression) was downloaded from NCBI Gene Expression Omnibus (GEO) public database (GSE156498) [13]. In this snRNA-seq dataset, nuclei were isolated from TA muscle of ΔEx51 *DMD^mut^* mice and WT mice at 4 weeks of age.

### 4.2. SnRNA-Seq Data Analysis

Seurat R package (V4.2) was used for downstream analytic procedures. Cells with extreme feature counts (<200 or >2500) and >20% reads with mitochondrial alignment were removed. Subsequently, we performed data normalization, high-variance feature identification, data scaling, and principal component analysis (PCA) using Seurat’s classic workflow. Then, the Harmony algorithm was used to correct the batch effects among samples. Next, dimensional reduction was performed using Uniform Manifold Approximation and Projection (UMAP) with the parameter “reduction” set as “harmony”. Seurat’s “FindNeighbors” and “FindClusters” functions were applied to the cell clustering analysis. MuSCs were annotated according to the MuSC lineage marker gene Paired Box 7 (Pax7) and the MuSC cluster was used for downstream analysis. MuSC cluster was further reclustered to generate six cluster subsets (0–5) under wide-type and DMD conditions. To identify differentially expressed genes (DEGs) between *DMD^mut^* MuSCs and control MuSCs, “FindMarkers” function under the default Wilcoxon rank sum test was applied to identify DEGs with avg_log2FC > 1 and *p*_val_adj < 0.05 as significant differential abundance. Volcano plots were generated using the R package “EnhancedVolcano” (V1.14).

### 4.3. Gene Ontology and Gene Set Enrichment Analysis

We applied the gseGO function from R/Bioconductor “clusterProfiler package” (V4.4.4) and org.Mmu.eg.db (V3.15) to perform gene ontology (GO) pathway enrichment analysis and Gene Set Enrichment Analysis (GSEA) of DEGs with default parameters. The terms with *p* values < 0.05 identified from GO pathway enrichment analysis were considered significant. GO enrichment analyses were visualized as bubble plots, and the network of most enriched terms was visualized by cnetplot function.

### 4.4. Single-Cell Metabolic Analysis

To discern the metabolic difference between *DMD^mut^* and control MuSCs in snRNA-seq datasets, we applied the “scMetabolism” package to quantify the metabolic activities of individual MuSCs in *DMD^mut^* versus wild-type control mice. Specifically, the method was set to “AUCells” and analyzed using the Kyoto Encyclopedia of Genes and Genomes (KEGG) metabolic gene sets.

### 4.5. Statistical Analysis

All statistical analyses and data presentation were performed using R program version 4.2.0.

## 5. Conclusions and Limitations

In conclusion, our snRNA-seq analysis demonstrated that MuSCs express the *dystrophin* gene. By comparing MuSCs from *DMD^mut^* mice and control mice, we identified critical target genes and signal pathways of *dystrophin* in MuSCs. Our findings indicate that the *dystrophin* mutation negatively affects the circulatory system process and blood circulation while promoting the activation of cell adhesion pathways. Furthermore, our snRNA-seq analysis highlighted differences in the metabolic pathways of MuSCs between *DMD^mut^* mice and control mice. This information adds to our understanding of the molecular changes occurring in DMD mutant MuSCs and their potential impact on muscle regeneration. However, this study has the following limitations:

(1) Our study primarily focuses on an “-omics” analysis of DMD function in MuSCs. It is indeed important to emphasize that the results of our bioinformatic analysis have not been experimentally validated, and further insights are required to fully assess their value. For example, our study reported the discovery of new LncRNAs and metabolic mechanisms associated with *DMD* mutation in MuSCs. However, none of these proposed mechanisms underwent validation;
(2) It is essential to acknowledge that the findings should be interpreted with caution, as the observed effects might be influenced by the early stage of the disease and the ongoing compensatory mechanisms. Future studies focusing on older animals or specifically investigating senescence-related pathways will provide further insights into the progressive nature of MuSC dysfunction in DMD.

## Figures and Tables

**Table 1:** Cluster-specific biomarkers.

Cluster	Gene
0	*GM26870 MT-CO3 MT-ATP6 MT-CYTB MT-ND2 MT-CO1 MT-ND4 MT-ND1 MT-CO2 MT-ND3*
1	*RORA HMCN2 CHODL LTBP1 CALCR CLMN KCNMA1 SOX5 BMP6 RCAN2*
2	*DIAPH3 MKI67 TOP2A KNL1 SDK1 LOCKD NEIL3 KIF20B CENPP CENPF*
3	*GM28653 KCNK13 TMEM178B KCNQ1OT1 FRMD4B RALGPS2 PAK3 DPP6 JAM3*
4	*CHODL NOTCH3 MDFIC CALCR BMP6 FOXP2 GULP1 CEP128 PDE3A KCNMA1*
5	*TRDN NEB TTN OBSCN MYH4 PDE4DIP RYR1 CMYA5 ATP2A1 NEAT1*

## Data Availability

Not applicable.
